# Supplementary material for: Ocean deoxygenation after the Sturtian Snowball
Source: Nat Commun. 2025 Jul 1;16:5618. doi: 10.1038/s41467-025-60700-w (PMC12219592; doi:10.1038/s41467-025-60700-w)
Supplement: Supplementary file 1 — Supplementary Information [file 41467_2025_60700_MOESM1_ESM.pdf]

Supplementary information for:

Ocean deoxygenation after the Sturtian Snowball

Kun Zhang [*], Susan H. Little, Alexander J. Dickson, Graham A. Shields

*Correspondence: kun-zhang@ucl.ac.uk

Supplementary information guide:

Supplementary Notes

Supplementary Figs. 1 to 10

Supplementary References

Additional supplementary file (separate file):

Supplementary Data (Excel file)

Supplementary Notes:

Geological, stratigraphic context and age model

The Zavkhan Terrane in western Mongolia is a late Neoproterozoic ribbon continent that was later embedded within the Central Asian Orogenic Belt[1,2]. The Neoproterozoic Tsagaan-Olom Group of the Zavkhan Terrane represents passive margin deposits and comprises the Cryogenian Maikhan-Uul, Taishir and Khongor Formations, and the Ediacaran Ol and Shuurgat Formations[3–5]. The Maikhan-Uul Formation was deposited during the Sturtian glaciation and is mainly composed of glaciogenic diamictites and siliciclastic rocks[3-7]. The base of the conformably overlying Taishir Formation has been dated at 659.0 ± 4.5 Ma with Re-Os geochronology[8], suggesting that it was probably deposited after the Sturtian deglaciation[9]. The Taishir Formation is 100–600 m thick, dominated by limestones deposited on an open marine homoclinal carbonate ramp, and is divided into four members (T1–T4)[5]. In the Taishir, Bayan, Uliastai and Tsagaan regions, the T1 member is generally dominated by micritic limestone, followed by predominantly grainstone in T2. The succeeding T3 mainly consists of thin-bedded micritic limestone in its lower part and bedded grainstone in its upper part. Ooids are relatively common throughout T2 and T3. The uppermost T4 is generally truncated or absent due to erosion, presumably related to glacial sea level fall, as it is overlain by the 0–50 m thick Marinoan-age diamictite of the Khongor Formation.

The studied samples of the Taishir Formation were collected at Tsagaan Gorge[3,7], which is around 8 km to the west of Uliastai Gorge section[10]. At Tsagaan Gorge, the base of the Taishir Formation is marked by several meters of pale gray, poorly laminated dusty microspar underlain by several decimeters of greenish and reddish shale[3,7]. This level is overlain by ~20 m of laminated micrite and ~10 m of non-laminated peloidal limestone (unit 16), above which there is ~20 m of less pure carbonate amid poor exposure (unit 17). This is followed by ~20 m of peloidal limestone and clotted thrombolite with a brecciated exposure surface at the top (unit 18). The overlying strata (unit 19) begin with non-laminated micrite with occasional oolites and transition into fine-bedded laminated limestones. There exists an unconformity at its top, which is considered to correspond to the Marinoan glaciation[3,11]. This succession has been divided into two sequences, i.e.,

sequence 3 (including units 16-18) and 4 (unit 19), and each represents a broadly shoaling upward sequence[6].

For this study, we correlated the Tsagaan section with the Uliastai Gorge and Khongor sections based on the carbon and strontium isotope chemostratigraphy (Fig. S1). T1 is characterised by rising $\delta^{13}C_{carb}$ from negative to positive values (mostly <+4‰) and rising $^{87}Sr/^{86}Sr$ values from ~0.7067 to ~0.7071, while $\delta^{13}C_{carb}$ and $^{87}Sr/^{86}Sr$ in T2 mostly plateau near +8‰ and 0.7071–0.7072, respectively[5]. The boundary between T2 and T3 is marked by a negative $\delta^{13}C_{carb}$ excursion of >10‰ (Taishir anomaly)[4]. Consequently, we correlate unit 16 with T1 given the similarly rising $\delta^{13}C_{carb}$ and $^{87}Sr/^{86}Sr$ values (Fig. S1). Considering the $^{87}Sr/^{86}Sr$ values of ~0.7071 and rising $\delta^{13}C_{carb}$ values approaching +8‰ in units 17 and 18, we suggest that the units could be correlated with T2. The Taishir excursion has not been identified at Tsagaan Gorge due in part to the relatively low-resolution of $\delta^{13}C_{carb}$ records, although a negative $\delta^{13}C_{carb}$ datapoint occurs in the middle of unit 19, underlying high $\delta^{13}C_{carb}$ values >+8‰. Because this is very similar to the records at Uliastai Gorge and Khongor (Fig. S1), this level could potentially mark the base of T3. However, this does not affect our study as the samples here span the lowermost ~120 m of the Taishir Formation and so are stratigraphically much lower than the T2-T3 boundary.

The age model for the Taishir Formation is not well resolved. Here the correlation of Maikhan-Uul diamictites with the Sturtian glaciation, similar $^{87}Sr/^{86}Sr$ values from the basal Taishir Formation in Mongolia and the basal Twitya Formation in Canada, and a Re-Os age of 659.0 ± 4.5 Ma near the base of the Taishir Formation lead us to place the base of the Taishir Formation at 661 Ma[3–5,9,11,12]. However, the age for the top of the Taishir Formation is loosely constrained due to a lack of radiometric ages from this interval and uncertainties related to the timing of the Marinoan glaciation onset. The synglacial Ghaub Formation in Namibia yielded a U-Pb zircon age of 639.29 ± 0.26 Ma[13], and the nonglacial Datangpo Formation in South China and Thorndike submember in Death Valley (USA) gave U-Pb zircon ages of 651.2 ± 0.64 Ma[14] and 651.69 ± 0.64 Ma[15], respectively. Thus, the present age constraints indicate that the Marinoan glaciation could have begun at c. 645 ± 5 Ma. Considering the erosion of T4 (possibly containing the Trezona anomaly), the initial Taishir Formation at Tsagaan might have been ~600 m thick. This gives an

estimation of the minimum and maximum constant sediment accumulation rates of 28.6 m/Ma and 54.5 m/Ma, respectively. Consequently, the studied interval may span a duration of ~2–4 Myr. Alternatively, the duration can be tested with Sr isotopes (see ref.[16] for an example of application). The seawater $^{87}Sr/^{86}Sr$ changed by ~0.0005 during the deposition of the studied interval. Assuming that the oceanic Sr residence time has not been radically different through the Neoproterozoic to the Phanerozoic and if we take the maximum change rate of $^{87}Sr/^{86}Sr$ during the Phanerozoic (i.e., 0.000192)[17], then the duration of the deposition could have been as short as 2.6 Myr. It should be noted that we are not aimed at quantifying the duration precisely, but our estimation provides the constraint that the deposition of studied interval spanned several million years and so was unlikely to have been affected by the meltwater plume (see the following discussion). Our estimation is also consistent with the age model of ref.[18] and so we invoked their age model A to facilitate the comparison with global data (Fig. 3). In addition to model A, ref.[18] proposed three alternative age models and demonstrated that the overall trends remain consistent regardless of the model used. We explored this here by employing the alternative age model D, which represents an extreme case of the non-glacial duration of c. 21 Myr. It appears that the trends do not change significantly (Fig. S2) and so this would not influence our interpretations.

Evaluating the potential influence of the meltwater plume

The global occurrence of cap dolostone after the Marinoan glaciation is characterised by tube-like structures and giant wave ripples, which was deposited during the transgressive system tract (TST)[19]. The deposition of these unique carbonates has been associated with the meltwater plume[20]. This hypothesis has been supported by carbonate Sr, Mg, and Li isotopes[21,22], and so this ‘plumeworld’ model has been extended to Sturtian cap carbonates. Considering that the studied interval was deposited in the aftermath of Sturtian deglaciation, it is important to evaluate whether these rocks were influenced by glacial meltwater during deposition. It should be noted that the TST is generally absent or highly condensed in non-glacial Cryogenian carbonates directly overlying Sturtian diamictites[19]. We also do not observe the sedimentary features similar to Marinoan cap carbonates in the studied section. On the other hand, the meltwater-affected Marinoan cap carbonates are characterised by unusually high $^{87}Sr/^{86}Sr$ values due to the highly

radiogenic feature of meltwater[21]. Similar phenomenon has been observed from the very basal part (~3 m) of the Twitya Formation in northwest Canada[12], suggesting the possible existence of locally preserved Sturtian cap carbonates. However, such high $^{87}Sr/^{86}Sr$ values are not observed from the basal part of the studied interval in Mongolia (Fig. 2), which are instead consistent with the values from Twitya carbonates above the highly radiogenic interval[12] and Rasthof carbonates[23]. These values are now widely regarded as representing the seawater composition[5,16,24,25]. Together, we suggest that the studied interval has not been affected by the meltwater during deposition.

Assessment of diagenetic effects on geochemical trends

Multiple geochemical proxies were extracted following established leaching protocols from samples with high carbonate contents (see Methods), which helps minimise contamination from non-carbonate phases. P readsorption during leaching, if any, could have decreased P/(Ca+Mg) values[26]. However, given the similar lithology and carbonate contents, we consider that this effect is unlikely to reverse the stratigraphic trend. We observe no significant covariations among most geochemical proxies and major element concentrations (Fig. S3). Although there seems a moderate covariation between $\delta^{238}U$ and Al contents, this relationship is strongly biased by one extremely negative $\delta^{238}U$ value with a U/Al ratio more than three orders of magnitude higher than the upper continental crust[27], indicating negligible detrital contamination. There is also a moderate covariation between [CAS] and TOC. However, it is unclear whether the covariation based on just five data points truly reflects causal relationships. Indeed, considering the relatively low and narrow range of organic matter content and absence of significant covariation between $\delta^{34}S_{CAS}$ and TOC, we regard the correlation as spurious due to insufficient data. The consistently low I/(Ca+Mg) values are also unlikely to result from contamination because non-carbonate phase dissolution would generally give rise to elevated concentrations[28]. Together, these lines of evidence confirm that the geochemical signals are not significantly affected by contamination during sample dissolution.

Petrographic observations reveal that the samples are dominated by fine-grained calcite microspar without any significant recrystallisation[7]. Together with high [Sr] (mostly >1000 ppm), low Mg/Ca (mostly <0.03), low Mn/Sr (mostly <0.2; Fig. S4), and pristine carbon and

strontium isotope ratios[7], the samples are considered well-preserved. We further assess the diagenesis using traditional geochemical diagenetic indicators such as $\delta^{18}O$, Mg/Ca, Mn/Sr, and Sr/Ca[29–31]. Although it is very likely that geochemical proxies respond differently from the diagenetic indicators to fluid-rock interactions, these typical indicators constitute a helpful framework to evaluate whether proxies are strongly altered by diagenesis. There are no significant covariations among most geochemical proxies and diagenetic indicators (Fig. S5). Nonetheless, we observe a moderate covariation between $\delta^{238}U$ and Mg/Ca. It has been shown that significant dolomitization (e.g., Mg/Ca > ~0.2) may decrease carbonate $\delta^{238}U$ values[32], whereas our samples characterised by low Mg/Ca ratios (<0.03) are unaffected by dolomitization. The negative covariation is apparently biased by the very negative $\delta^{238}U$ value, removal of which causes the covariation to disappear. We also find a strong positive covariation between Sr/Ca and $\delta^{34}S_{CAS}$ (Fig. S5), which could be spurious due to limited $\delta^{34}S_{CAS}$ data. Alternatively, if we consider the covariation to be genuine, the positive covariation between Sr/Ca and $\delta^{34}S_{CAS}$ implies that the relatively lower $\delta^{34}S_{CAS}$ values may be the result of diagenetic alteration. This is because Sr/Ca is generally lower in altered carbonates due to the loss of Sr during diagenesis. Nevertheless, it should be noted that we do not observe significant covariations between Mn/Sr, $\delta^{18}O$ and Sr/Ca, which are expected to covary during diagenesis [29]. In addition to diagenesis, the variation of carbonate Sr/Ca can also be affected by other factors such as seawater Sr/Ca. Hence, the observed covariation may also represent closely linked environmental factors that caused the simultaneous variation of $\delta^{34}S_{CAS}$ and Sr/Ca. Regardless of the reason behind this covariation, it appears that the extremely high $\delta^{34}S_{CAS}$ are unlikely to be significantly altered. Consequently, this would not influence our interpretation of euxinia in the lower part (see the main text).

High Sr concentrations of samples indicate that they were likely originally aragonites and so could have experienced neomorphism during early diagenesis. Given that diagenetic processes generally lead to iodine loss from carbonate rocks[33], it is plausible that the carbonate I/(Ca+Mg) values could have been lowered to some extent during the neomorphism. Therefore, the low I/(Ca+Mg) ratios in our samples may be attributed to diagenesis in addition to precipitating in reducing water column or near the shallow oxycline. Nevertheless, despite the diagenetic alteration of I/(Ca+Mg), the geochemical

trends of other proxies are not necessarily affected concomitantly owing to the high sensitivity of I/(Ca+Mg) to diagenesis[33,34] (see also discussion below).

The influence of diagenesis on REE is most easily discernible from the shale-normalised REE distribution pattern[35–37]. The appearance of a seawater-like REE pattern characterised by positive La and Y anomalies, heavy REE enrichment, and absence of middle REE enrichment indicate the preservation of a primary seawater REE signature. Since nearly all samples show moderate positive La and Y anomalies, discrimination of non-seawater-like from seawater-like REE patterns here is mainly based on $Pr_N/Yb_N>1$, $Sm_N/Yb_N>1$, and BSI>1.2. It appears that some samples from the lower part of the succession are characterised by non-seawater-like REE patterns (Fig. S6), which have been attributed to early diagenesis[37]. It is thus necessary to assess whether other geochemical proxies are also altered in conjunction with the distortion of REE. Considering that $\delta^{238}U$ of Fe-Mn oxides and organic matter is ~0.2‰ lighter than the seawater[38,39], remobilized U from these sources, if incorporated during early diagenesis, should shift carbonate $\delta^{238}U$ to lower values as shown for Paleogene carbonates[40]. However, we observe no distinct $\delta^{238}U$ values between neighbouring samples with seawater-like and non-seawater-like REE patterns. Our finding is thus consistent with the emerging consensus that there is little evidence that REE and U would be tightly coupled during early diagenesis[37,41,42]. Additionally, while $\delta^{66}Zn$ of Fe-Mn oxides is heavier than seawater by ~0.5‰, the organic matter $\delta^{66}Zn$ on the continental shelf is possibly close to or slightly lower than the seawater $\delta^{66}Zn$[43,44]. As such, incorporation of the remobilized Zn would likely intend to increase carbonate $\delta^{66}Zn$ values, instead of producing a continuous decreasing stratigraphic trend of $\delta^{66}Zn$ observed in the lower part, from which most non-seawater-like REE patterns are revealed. Comparable $\delta^{66}Zn$ values are also obtained from neighbouring samples with contrasting REE patterns. On the other hand, while it remains possible that the carbonate P/(Ca+Mg) values may have been decreased to some extent during the aragonite-to-calcite neomorphism probably due to a relatively higher partition coefficient of phosphate in aragonite relative to calcite[26], the consistently high Sr concentrations and absence of covariation between P/(Ca+Mg) and Sr/Ca suggest that it does not affect the first-order stratigraphic trend observed here. Conversely, organic matter remineralization and/or reductive dissolution of iron oxides could elevate pore

water phosphate concentrations, which may potentially increase carbonate P/(Ca+Mg) values[45]. However, P/(Ca+Mg) values remain largely constant throughout the succession (Fig. 2), exhibiting no significant difference between neighbouring samples with seawater-like and non-seawater-like REE patterns. Together with the absence of significant covariation between $\delta^{238}U$, $\delta^{66}Zn$, P/(Ca+Mg) and REE shape parameters (Fig. S7), it is thus unlikely that the geochemical proxies have been altered simultaneously with the distortion of REE patterns.

We consider the response of CAS proxy to early diagenesis here. Microbial sulfate reduction during early diagenesis could elevate $\delta^{34}S_{CAS}$ by enriching the residual pore fluid sulfate with heavy ^{34}S through Rayleigh distillation, while pyrite oxidation could decrease $\delta^{34}S_{CAS}$ by introducing ^{32}S enriched sulfate. The extent to which $\delta^{34}S_{CAS}$ deviates from the seawater value during diagenesis depends on the sulfate concentration of the original carbonate, the sulfate concentration and $\delta^{34}S$ composition of the diagenetic fluid, the relative rates of carbonate recrystallisation and sedimentation, and the openness of the fluid-rock system[46–50]. It is expected that seawater $\delta^{34}S$ values are more likely to be preserved in carbonates with high initial sulfate concentrations, or in carbonates with low sulfate concentrations yet interacting with sulfate depleted fluid or with unevolved fluid with respect to $\delta^{34}S$[51]. The relationship between [CAS] and $\delta^{34}S_{CAS}$ has been proposed to discern the origin of $\delta^{34}S_{CAS}$ variability[52,53]. We observe a moderate negative covariation between the two parameters (Fig. S8), but as discussed above the causal relationship remains contentious due to the limited data points. It might be caused by sulfate reduction during early diagenesis, whereby a large sulfur isotope fractionation during sulfate reduction would only be expressed under a moderate sulfate reservoir[54,55]. Meanwhile, the residual pore water sulfate incorporated into recrystallized and/or authigenic carbonates can achieve high enough values to drive bulk $\delta^{34}S_{CAS}$ to higher values. However, it has been proposed that the post-Sturtian ocean was depleted in sulfate as evidenced by high $\delta^{34}S_{CAS}$ and $\delta^{34}S_{py}$, generally low $\Delta^{34}S$ of <10‰[56,57] and extremely low [CAS] in our samples and coeval carbonates[57]. If we assume that the outlier in the lower part, characterised by relatively high [CAS] but low $\delta^{34}S_{CAS}$ of 44‰, represents the primary isotope composition, the Rayleigh distillation modelling reveals that nearly 80-90% sulfate in the pore fluid would have been consumed to produce high

sulfate $\delta^{34}S$ values (60-70‰) observed in our samples (Fig. S9). Note that a constant isotope fractionation of 10‰ is assumed, but in reality, the offset should have been decreased with decreasing pore water sulfate concentrations[54,55]. Therefore, with nearly depleted pore water sulfate, the modelling result supports that the high $\delta^{34}S_{CAS}$ values are unlikely to be explained by early diagenesis. Alternatively, the negative covariation may suggest pyrite oxidation during either laboratory extraction or diagenesis[58,59]. This may be particularly relevant for the outlier in the lower part, which shows the highest CAS concentration of 17 ppm and exhibits the greatest deviation of ~20‰ from adjacent samples. Such amplified variability within a short stratigraphic interval of 3 m could indicate pyrite oxidation, although it remains possible that $\delta^{34}S_{CAS}$ could exhibit great fluctuations in a low-sulfate ocean. There is little evidence for pyrite oxidation during early diagenesis as the sample is characterised by fine-grained calcite with finely laminated structure, suggesting that it was deposited in a low-energy reducing seawater[6,11] (also see the main text). This leads us to consider that the sample could have been affected by pyrite oxidation during CAS extraction despite the rigorous cleaning steps. Thus, the sample is excluded from further discussion in the main text. The other samples show relatively consistent [CAS] and exhibit a stratigraphic trend of declining $\delta^{34}S_{CAS}$ similar to coeval strata[57,60]. Taken together, we consider that $\delta^{34}S_{CAS}$ is unlikely to have been significantly altered during early diagenesis except for the lone sample that has potentially been influenced by pyrite oxidation.

Local redox conditions may also have played a role in affecting carbonate $\delta^{238}U$ values. This is because authigenic accumulation of U(IV) in early diagenetic reducing pore fluids could lead to elevated carbonate [U] and $\delta^{238}U$ values due to the preferential reduction of ^{238}U to insoluble U(IV)[32,40,41]. This may be especially relevant for the lower part, which shows consistently positive Ce anomalies indicative of a locally reducing water column (also see the main text). Coincidently, the carbonate [U] is relatively higher in the lower part (mean 1.05 ppm) than the upper part (mean 0.54 ppm), implying that some authigenic U(IV) accumulation may have occurred during deposition of the lower part. Nevertheless, we do not observe the covariation between [U] and $\delta^{238}U$ (Fig. S8), which is commonly invoked to infer the influence of authigenic U(IV) enrichment on carbonate uranium isotope values[40,41]. This suggests that despite the possible minor authigenic

enrichment, the stratigraphic trend of $\delta^{238}U$ is not significantly affected, possibly due to the relatively strong resistance of aragonite to authigenic U(IV) incorporation because of its high initial U concentration[41,61]. The influence of early diagenetic reducing pore fluid on carbonate $\delta^{66}Zn$ is unclear at this stage. If the pore fluid is sulfidic (though less likely; see the main text), Zn sulfide species would preferentially incorporate light Zn[62], leaving the residual Zn pool and perhaps carbonate $\delta^{66}Zn$ isotopically heavier, but this is not observed (Fig. 2). Lastly, if the geochemical signals are regulated by reducing pore fluids during the early diagenesis, one would expect that their variations follow the array of pore water chemistry[45] such as the concomitantly elevated P/(Ca+Mg), $\delta^{34}S_{CAS}$, $\delta^{238}U$[45], which is, however, inconsistent with the observed trends here.

Collectively, following from these observations, we conclude that while we cannot rule out diagenetic proxy alteration, the first-order stratigraphic trends observed here are, by and large, not governed by diagenetic processes but instead reflect changes in seawater composition. We explore the possible cause-and-effect relationships of the geochemical trends in the main text and acknowledge that more studies from coeval strata would be necessary to test our hypothesis.

Estimation of the post-Sturtian open ocean $\delta^{34}S_{CAS}$

The average $\delta^{34}S_{CAS}$ of 63‰ in the lower part is among the highest values reported for the Cryogenian Period and probably throughout Earth history. The value is significantly higher than the previous estimate of open ocean sulfate $\delta^{34}S$, which was around 30-40‰[57,60,63]. The discrepancy might simply reflect the spatiotemporal heterogeneity of seawater sulfate $\delta^{34}S$ in a low-sulfate ocean. More critically, the timing of anhydrite deposition in the Tapley Hill Formation and replacive barites in the Bonahaven Dolomite Formation are poorly constrained but likely did not form during the immediate aftermath of Sturtian deglaciation[60,63]. Therefore, similar to the pyrite $\delta^{34}S$ records that exhibit a broadly declining trend during the nonglacial Cryogenian[18], their relatively lower values may reflect the lower seawater sulfate $\delta^{34}S$ long after Sturtian deglaciation. In this regard, we note that our $\delta^{34}S_{CAS}$ from the top of the succession is comparable to their data. Lastly, owing to the extremely low [CAS], the primary $\delta^{34}S_{CAS}$ could be easily masked by pyrite oxidation during laboratory extraction. A simple endmember mixing model (i.e., pristine

CAS and pyrite-oxidation derived sulfate) illustrates that the contamination from a small amount of pyrite oxidation can lead to markedly decreased $\delta^{34}S_{CAS}$ values (Fig. S10). Hence, the relatively low $\delta^{34}S_{CAS}$ in the Rasthof Formation could be accounted for by insufficient cleaning with only a single rinse of a 5.25% bleach solution before CAS extraction[57]. Overall, our new dataset suggests that the sulfate $\delta^{34}S$ value of open ocean waters in the immediate wake of Sturtian deglaciation was probably higher than most of the coeval pyrite $\delta^{34}S$ records that are mainly in the range of 0-60‰ (see ref.[18] for a recent compilation). If true, the so-called superheavy pyrite during this time interval is probably not as significant as previously envisaged and so easier to reconcile, while we note that more $\delta^{34}S_{CAS}$ data obtained with a robust leaching protocol would be needed to evaluate the global average of seawater sulfate $\delta^{34}S$.

Control of sea-level change on stratigraphic $\delta^{238}U$ and $\delta^{66}Zn$ variations

The anomalously low $\delta^{238}U$ values have previously been accounted for by water column U reduction[64] because this may allow the full expression of isotope fractionation to be recorded[65]. If this was the case, $\delta^{238}U$ would be expected to increase during shoaling of a redoxcline[66], which is contrary to the observed trend in our dataset (Fig. 2). This may refute the operation of water column U reduction, similar to the observation in the Black Sea[67]. Alternatively, it may indicate that the local water column was not reducing enough (non-euxinic) to trigger U reduction, although iron speciation data strongly suggest the presence of euxinic seawater elsewhere[18]. On the other hand, ref.[68] suggested that platform carbonate $\delta^{238}U$ values tend to be higher during the period of high sea-level due to a greater extent of authigenic U enrichment. They also indicated that the mixing of platform-derived carbonates driven by sea-level change may also increase $\delta^{238}U$ values, which causes the covariation of Sr/Ca and $\delta^{238}U$. However, as discussed above, authigenic U enrichment does not affect the stratigraphic trend of $\delta^{238}U$. There is also no covariation between $\delta^{238}U$ and Sr/Ca in the samples (Fig. S5). Therefore, the relative sea-level and local redox changes cannot account for the $\delta^{238}U$ stratigraphic variations in this case. The decreasing $\delta^{238}U$ is also unlikely to be induced by enhanced weathering as evidenced by the dramatic rise of $^{87}Sr/^{86}Sr$ (Fig. 2). This is because the uranium isotope composition of riverine input likely remained relatively constant after the Great Oxidation Event[69], while enhanced weathering rates would be more likely to cause a transient

positive excursion[10] rather than the observed decreasing trend.

The stratigraphic variation of δ^{66}Zn in the studied succession is unlikely to have been impacted by basin restriction as the Taishir Formation at Tsagaan Gorge was deposited on an open carbonate ramp[5]. The change of relative sea-level and local redox conditions also cannot account for the δ^{66}Zn stratigraphic variation. First, although dissolved δ^{66}Zn gradually increases across the euxinic redoxcline due to the preferential incorporation of isotopically light Zn by zinc-sulfide species[62], the locally non-sulfidic condition for the lower part indicates that the decreasing δ^{66}Zn with shallowing seawater is not caused by the change in Zn speciation. The association of relatively stable δ^{66}Zn values followed by a rise in the lower part is also distinct from observations of modern anoxic water columns. Notwithstanding the sea-level change with shallowing or across the sequence boundary, δ^{66}Zn values largely exhibit only a modest increase in the upper part deposited under the locally oxic conditions (Fig. 2), refuting any dominant control by sea-level change. On the other hand, it is hard to precisely pinpoint the causes for the variation of stratigraphic [Zn], which broadly increases throughout the succession but drops dramatically across the sequence boundary. The gradually increasing [Zn] with shallowing is in contrast to [Zn] variation with water depth in modern oceans. Nevertheless, irrespective of the exact reasons for the stratigraphic variation of [Zn], it appears to have no systematic influence on δ^{66}Zn (Fig. S8). Therefore, the δ^{66}Zn stratigraphic change is mostly ascribed to secular variations in the seawater Zn isotope composition.

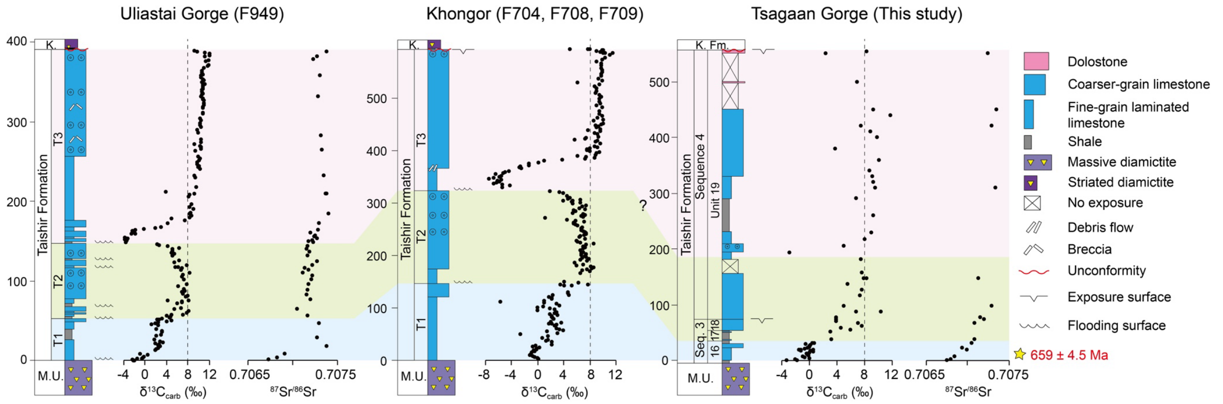

Fig. S1. Regional correlation of the Taishir Formation in the Zavkhan Terrane. Dashed lines indicate $\delta^{13}C_{carb}$ of +8‰. The stratigraphic column of the Uliastai section is modified after ref.[5]. The $\delta^{13}C_{carb}$ and $^{87}Sr/^{86}Sr$ data are from ref.[57] and ref.[5], respectively. The stratigraphic

column and $\delta^{13}C_{carb}$ data of the Khongor section are from ref.[4]. The member division of the two sections is from ref.[5]. The stratigraphic column, $\delta^{13}C_{carb}$ and $^{87}Sr/^{86}Sr$ data of the Tsagaan Gorge section are from ref.[7]. The yellow star marks the Re–Os age of organic-rich limestones from ref.[8].

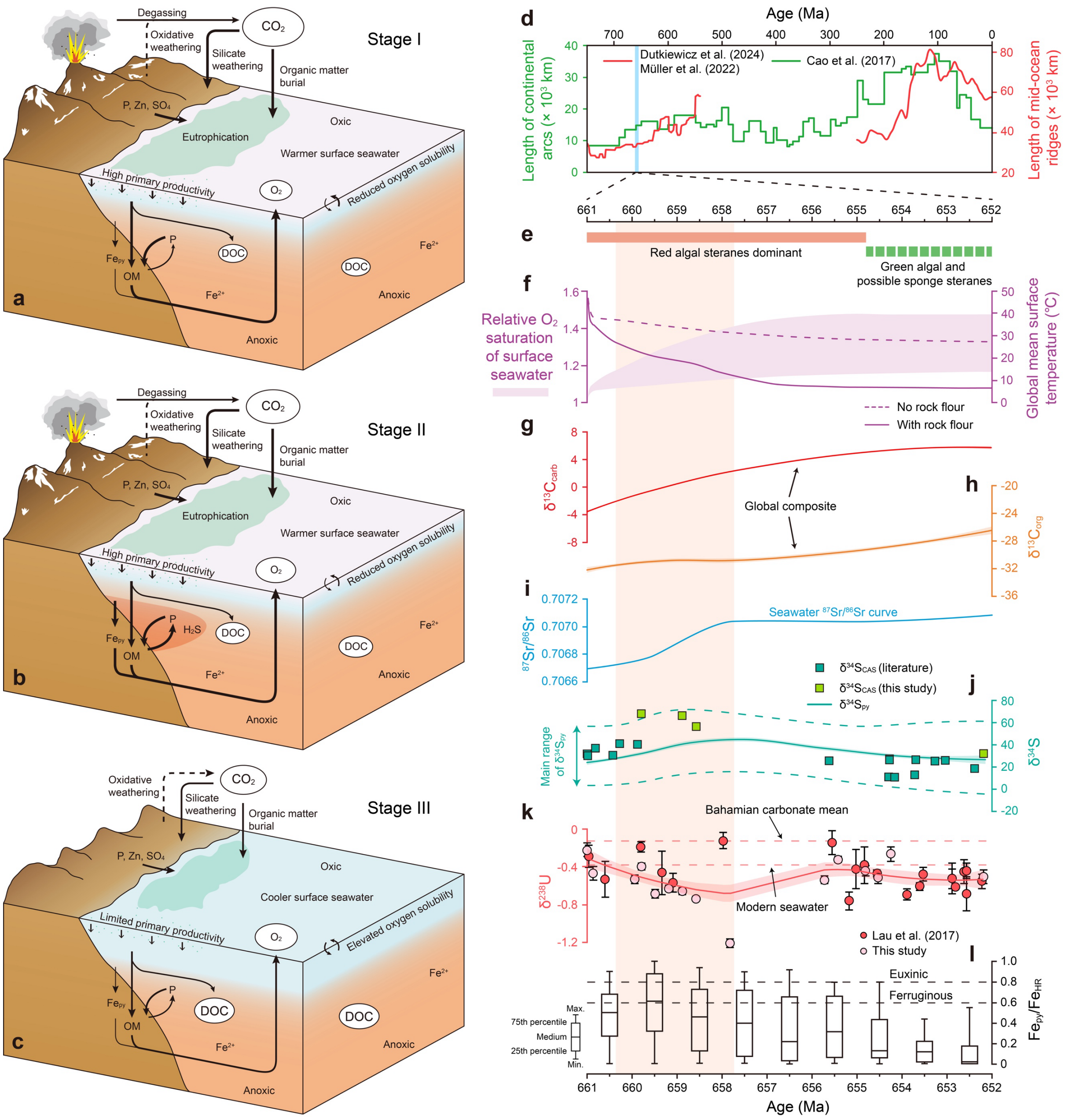

Fig. S2. Conceptual model and biogeochemical indicators following the Sturtian deglaciation. **a,** Vigorous weathering induced eutrophication after the Sturtian deglaciation. **b,** Expansion of euxinia on productive margins owing to excess of H_2S over iron. **c,** Continuous atmospheric CO_2 drawdown led to subdued weathering, cooling and shallow seawater oxygenation. In (**a-c**), the thicknesses of arrows indicate the relative

magnitude of fluxes, and the sizes of ellipses represent the relative sizes of reservoirs. **d,** Global length of continental arcs[71] and mid-ocean ridges[72,73]. The blue band indicates the studied interval. **e,** Occurrences of biomarkers[18]. **f,** Possible evolution of relative O_2 saturation of surface seawater and global mean surface temperature[18]. **g-l,** Compilation of carbon, strontium, sulfur, uranium isotopes and iron speciation records (modified after ref.[18]). Solid lines in **g,h,j,k** represent the LOESS (locally estimated scatterplot smoothing) fitting curves, and the shaded area represents 1σ (68%) confidence interval. The dashed line in **j** indicates the major range of $\delta^{34}S_{py}$. In **l**, each boxplot represents the distribution of global Fe_{py}/Fe_{HR} data in each one-million-year time bin. The light shade of pale red band indicates the second stage. The age model of **e-l** is based on model D of ref.[18]. Abbreviations: OM, organic matter burial; Fe_{py}, pyrite burial; DOC, dissolved organic carbon pool; Min., minimum; Max., maximum.

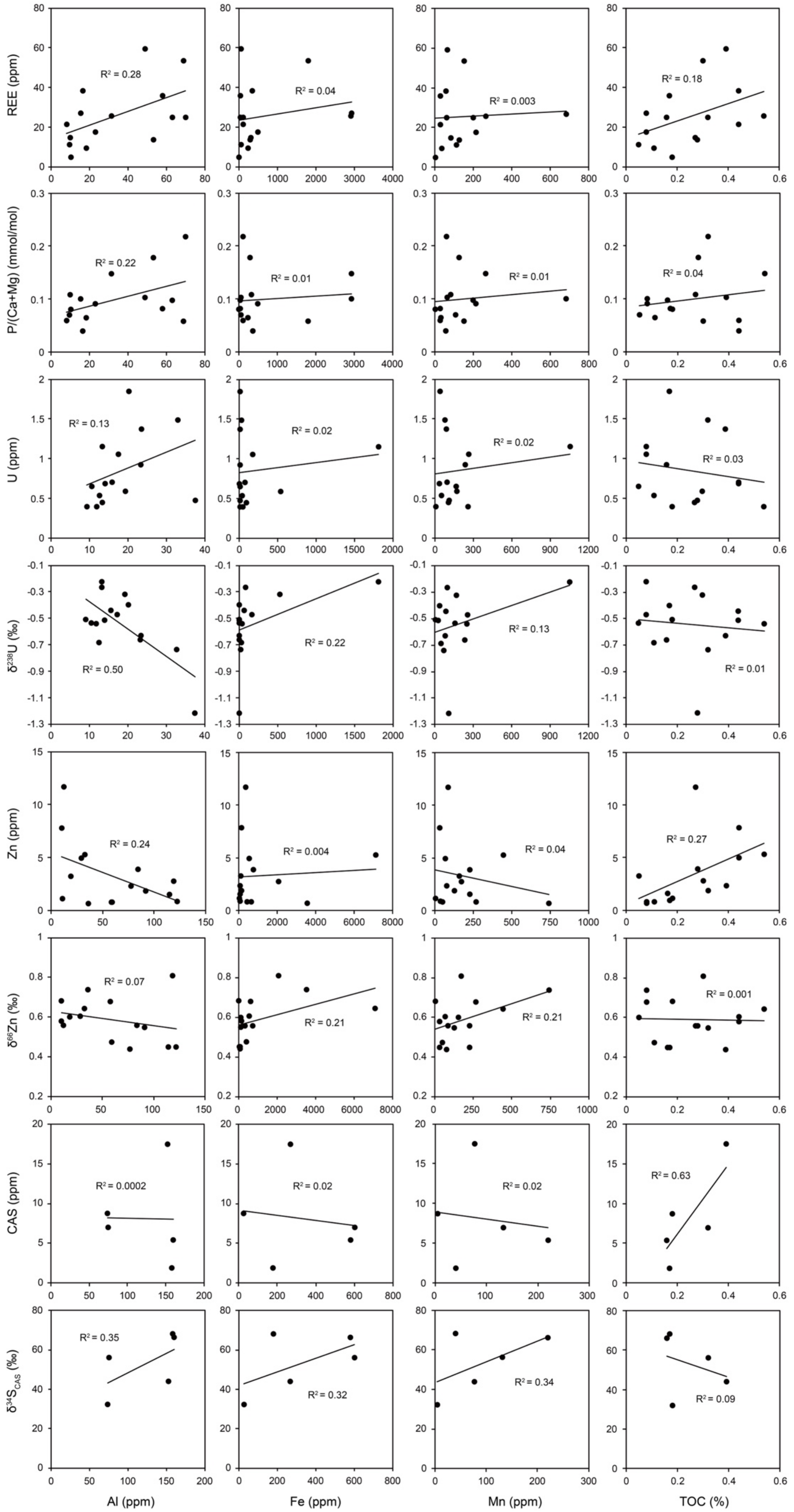
REE (ppm)
P/(Ca+Mg) (mmol/mol)
U (ppm)
δ238U (‰)
Zn (ppm)
δ66Zn (‰)
CAS (ppm)
δ34SCAS (‰)
Al (ppm)
Fe (ppm)
Mn (ppm)
TOC (%)
R² = 0.28
R² = 0.04
R² = 0.003
R² = 0.18
R² = 0.22
R² = 0.01
R² = 0.01
R² = 0.04
R² = 0.13
R² = 0.02
R² = 0.02
R² = 0.03
R² = 0.50
R² = 0.22
R² = 0.13
R² = 0.01
R² = 0.24
R² = 0.004
R² = 0.04
R² = 0.27
R² = 0.07
R² = 0.21
R² = 0.21
R² = 0.001
R² = 0.0002
R² = 0.02
R² = 0.02
R² = 0.63
R² = 0.35
R² = 0.32
R² = 0.34
R² = 0.09

Fig. S3. Cross plots of geochemical proxies against Al, Fe, Mn and TOC contents. TOC data are from ref.[7].

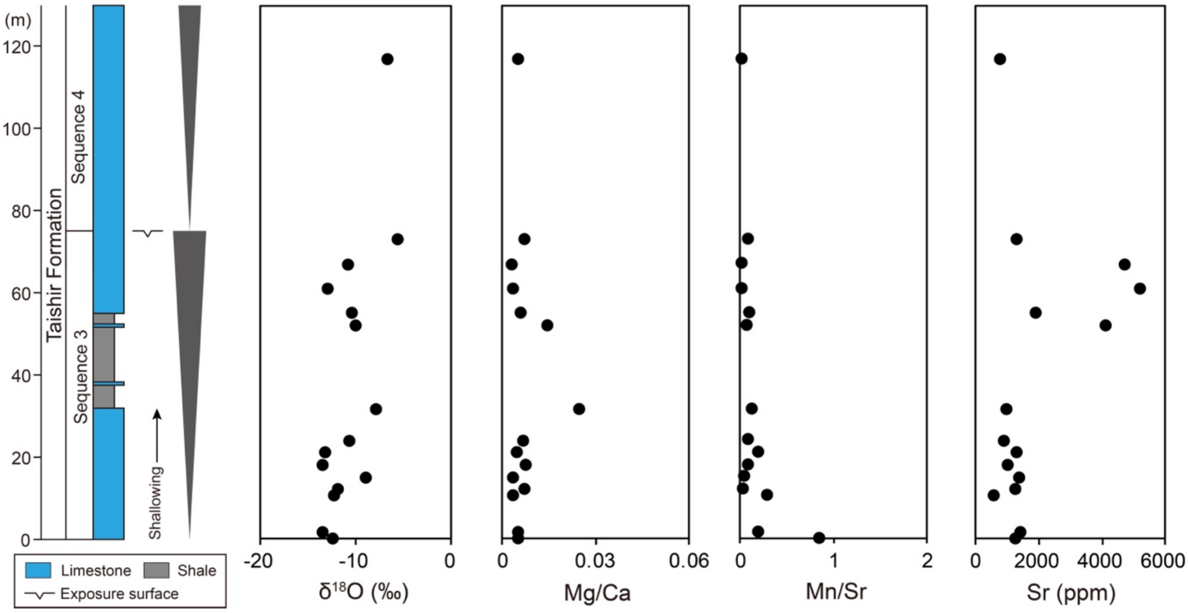

Fig. S4. Chemostratigraphic profiles of carbonate $\delta^{18}O$, Mg/Ca, Mn/Sr ratios and Sr concentrations for the studied succession from the lower part of Taishir Formation at Tsagaan Gorge. $\delta^{18}O$ data are from ref.[7].

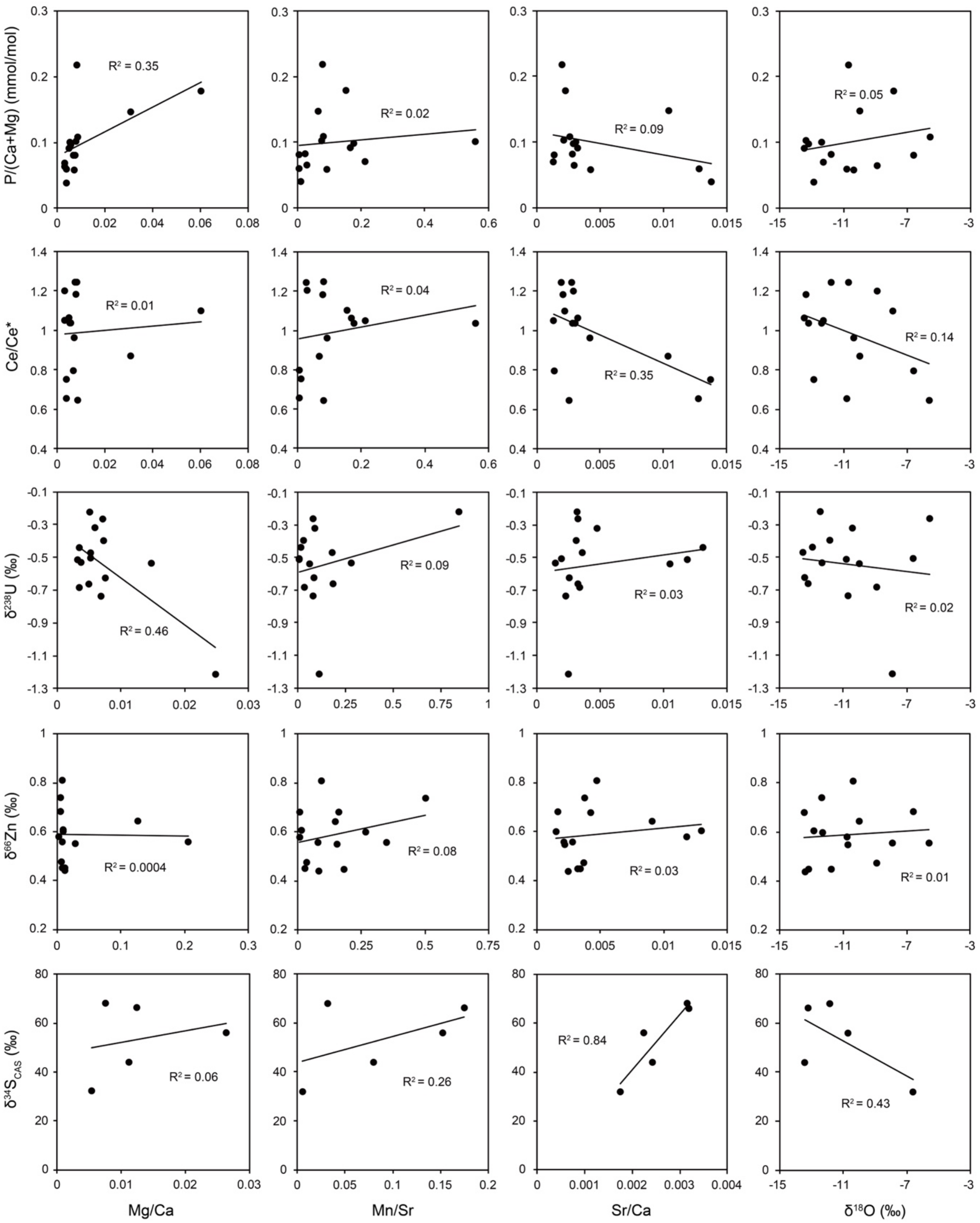

Fig. S5. Cross plots of geochemical proxies against commonly used diagenetic indicators. $\delta^{18}O$ data are from ref.[7].

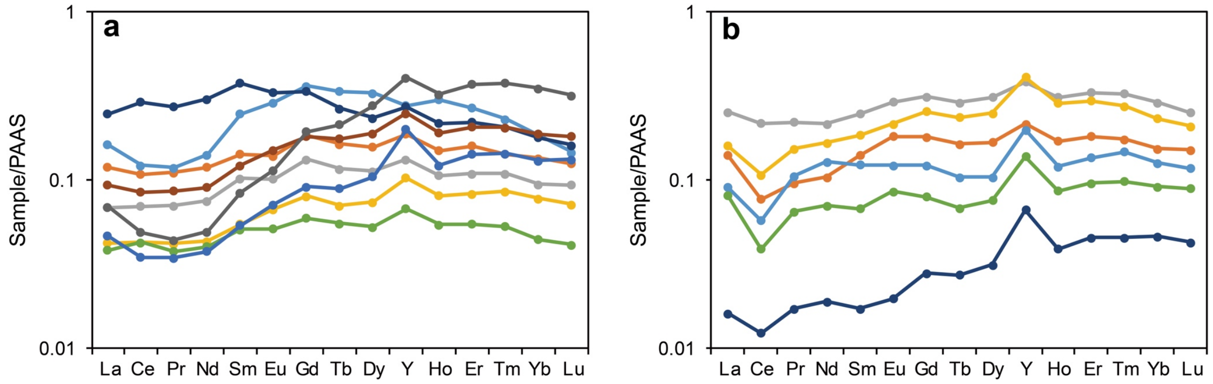

Fig. S6. REE patterns for the (a) lower part (0–32 m) and (b) upper part (50-120 m) of the studied succession of the Taishir Formation.

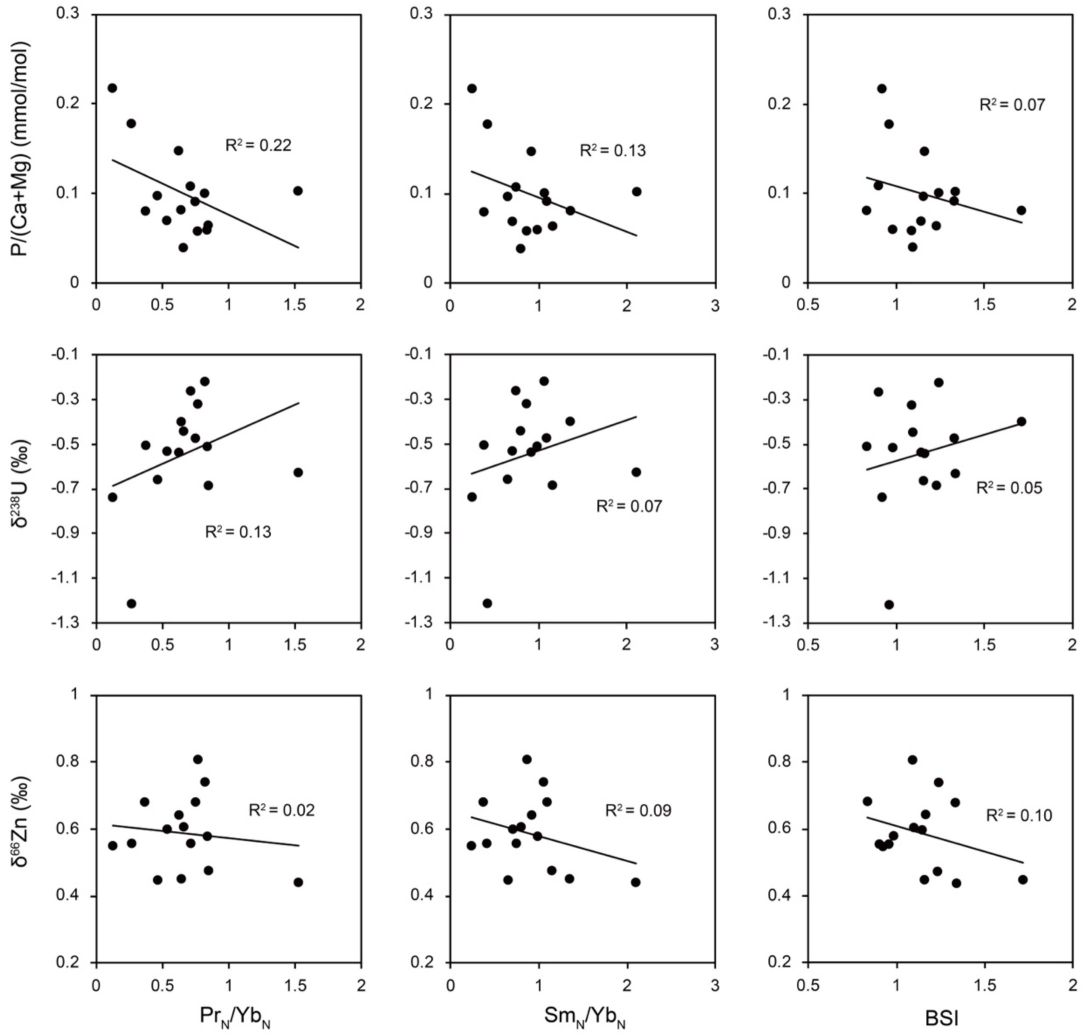

Fig. S7. Cross plots of P/(Ca+Mg), $\delta^{238}U$, and $\delta^{66}Zn$ against REE shape parameters including Pr_N/Yb_N, Sm_N/Yb_N, and BSI.

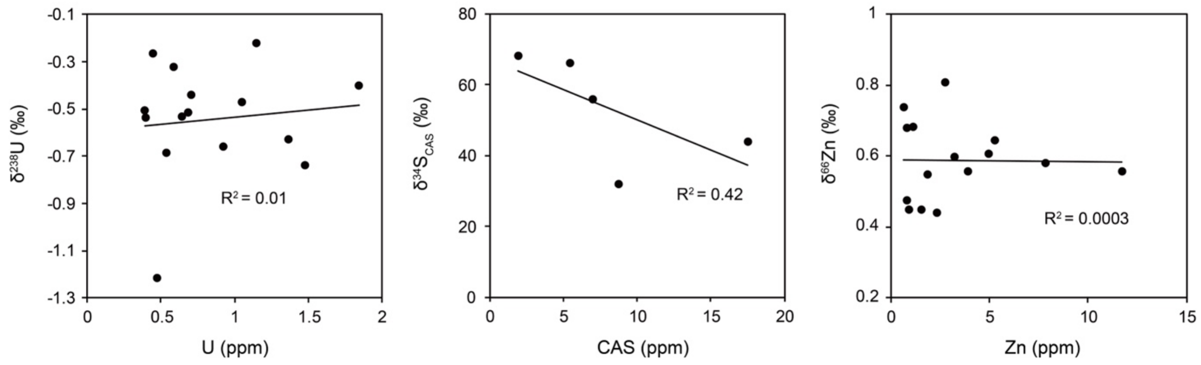

Fig. S8. Cross plots of $\delta^{238}U$ vs. [U], $\delta^{34}S_{CAS}$ vs. [CAS], and $\delta^{66}Zn$ vs. [Zn] for studied samples.

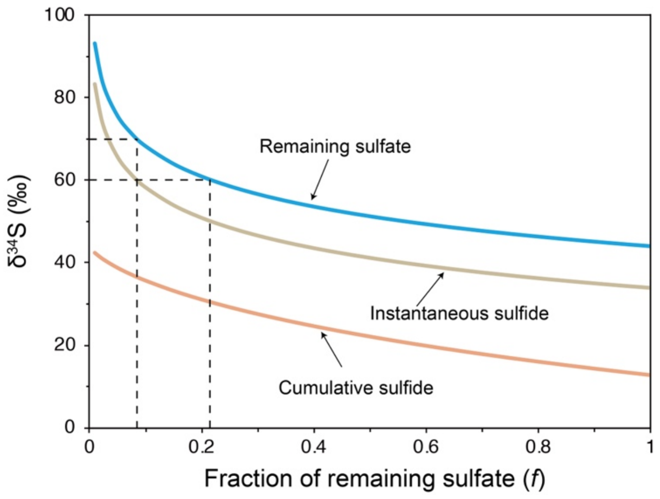

Fig. S9. Pore water $\delta^{34}S$ evolution via Rayleigh distillation assuming a constant isotope fractionation of 10‰.

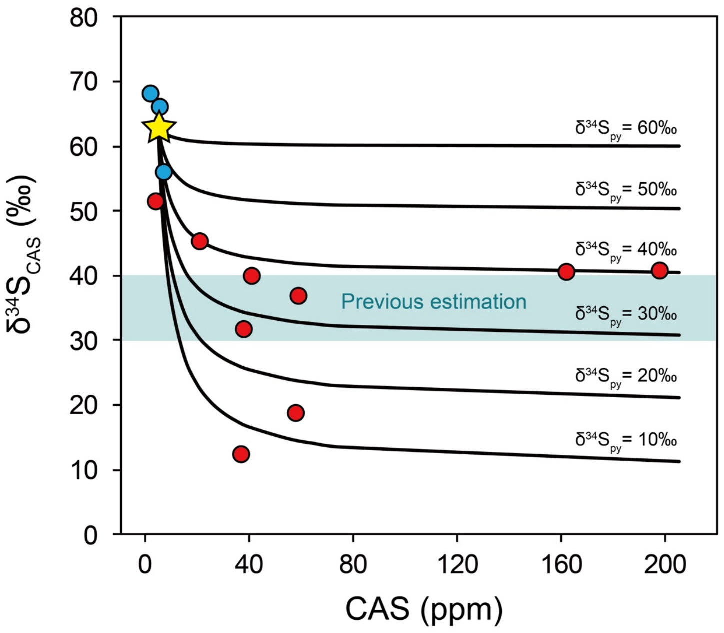

Fig. S10. Simple endmember mixing between primary carbonate-associated sulfate and pyrite oxidation induced sulfate. The figure is illustrative only to visualise the susceptibility of low [CAS] samples to pyrite oxidation. The colour band represents the previous estimation of open ocean sulfate $\delta^{34}S$ during the Cryogenian nonglacial interval. Contour lines indicate the mixing of pure CAS endmember with pyrite-derived sulfate with different $\delta^{34}S_{py}$ assuming negligible sulfur isotope fractionation during oxidation. Yellow star denotes the postulated pristine CAS endmember, represented by the average of the low part of our studied succession (blue circles). Red circles represent data from the lower part of the Rasthof Formation in Namibia[57].

Supplementary References:

1. Windley, B. F., Alexeiev, D., Xiao, W., Kröner, A. & Badarch, G. Tectonic models for accretion of the Central Asian Orogenic Belt. *J. Geol. Soc. London.* **164**, 31–47 (2007).
2. Bold, U. *et al.* Effect of dolomitization on isotopic records from Neoproterozoic carbonates in southwestern Mongolia. *Precambrian Res.* **350**, 105902 (2020).
3. Brasier, M. D., Shields, G., Kuleshov, V. N. & Zhegallo, E. A. Integrated chemo- and biostratigraphic calibration of early animal evolution: Neoproterozoic–early Cambrian of southwest Mongolia. *Geol. Mag.* **133**, 445–485 (1996).
4. Macdonald, F. A., Jones, D. S. & Schrag, D. P. Stratigraphic and tectonic implications of a newly discovered glacial diamictite-cap carbonate couplet in southwestern Mongolia. *Geology* **37**, 123–126 (2009).
5. Bold, U. *et al.* Neoproterozoic stratigraphy of the Zavkhan terrane of Mongolia: The backbone for Cryogenian and early Ediacaran chemostratigraphic records. *Am. J. Sci.* **316**, 1–63 (2016).
6. Lindsay, J. F. *et al.* Facies and sequence controls on the appearance of the Cambrian biota in southwestern Mongolia: Implications for the Precambrian-Cambrian boundary. *Geol. Mag.* **133**, 417–428 (1996).
7. Shields, G. A., Brasier, M. D., Stille, P. & Dorjnamjaa, D. Factors contributing to high $\delta^{13}C$ values in Cryogenian limestones of western Mongolia. *Earth Planet. Sci. Lett.* **196**, 99–111 (2002).
8. Rooney, A. D., Strauss, J. V., Brandon, A. D. & Macdonald, F. A. A Cryogenian chronology: Two long-lasting synchronous neoproterozoic glaciations. *Geology* **43**, 459–462 (2015).
9. Rooney, A. D., Yang, C., Condon, D. J., Zhu, M. & Macdonald, F. A. U-Pb and Re-Os geochronology tracks stratigraphic condensation in the Sturtian snowball Earth aftermath. *Geology* **48**, 625–629 (2020).
10. Lau, K. V., Macdonald, F. A., Maher, K. & Payne, J. L. Uranium isotope evidence for temporary ocean oxygenation in the aftermath of the Sturtian Snowball Earth. *Earth Planet. Sci. Lett.* **458**, 282–292 (2017).
11. Shields, G., Stille, P., Brasier, M. D. & Atudorei, N. Stratified oceans and

oxygenation of the late Precambrian environment: a post glacial geochemical record from the Neoproterozoic of W. Mongolia. *Terra Nov.* **9**, 218–222 (1997).

12. Rooney, A. D. *et al.* Re-Os geochronology and coupled Os-Sr isotope constraints on the Sturtian snowball Earth. *Proc. Natl. Acad. Sci. U. S. A.* **111**, 51–56 (2014).
13. Prave, A. R., Condon, D. J., Hoffmann, K. H., Tapster, S. & Fallick, A. E. Duration and nature of the end-Cryogenian (Marinoan) glaciation. *Geology* **44**, 631–634 (2016).
14. Ma, X. *et al.* Geochronological constraints on Cryogenian ice ages: Zircon U Pb ages from a shelf section in South China. *Glob. Planet. Change* **222**, 104071 (2023).
15. Nelson, L. L. *et al.* Geochronological constraints on Neoproterozoic rifting and onset of the Marinoan glaciation from the Kingston Peak Formation in Death Valley, California (USA). *Geology* **48**, 1083–1087 (2020).
16. Zhou, Y. *et al.* Reconstructing Tonian seawater 87Sr/86Sr using calcite microspar. *Geology* **48**, 462–467 (2020).
17. McArthur, J. M., Howarth, R. J. & Shields, G. A. Strontium isotope stratigraphy. *Geol. Time Scale 2012* 127–144 (2012) doi:10.1016/B978-0-444-59425-9.00007-X.
18. Bowyer, F. T. *et al.* Biological diversification linked to environmental stabilization following the Sturtian Snowball glaciation. *Sci. Adv.* **9**, eadf9999 (2023).
19. Hoffman, P. F. *et al.* Snowball Earth climate dynamics and Cryogenian geology-geobiology. *Sci. Adv.* **3**, e1600983 (2017).
20. Shields, G. A. Neoproterozoic cap carbonates: A critical appraisal of existing models and the plumeworld hypothesis. *Terra Nov.* **17**, 299–310 (2005).
21. Liu, C., Wang, Z., Raub, T. D., Macdonald, F. A. & Evans, D. A. D. Neoproterozoic cap-dolostone deposition in stratified glacial meltwater plume. *Earth Planet. Sci. Lett.* **404**, 22–32 (2014).
22. Gan, T. *et al.* Lithium isotope evidence for a plumeworld ocean in the aftermath of the Marinoan snowball Earth. *Proc. Natl. Acad. Sci.* **121**, 2017 (2024).
23. Yoshioka, H., Asahara, Y., Tojo, B. & Kawakami, S. ichi. Systematic variations in C, O, and Sr isotopes and elemental concentrations in neoproterozoic carbonates in Namibia: Implications for a glacial to interglacial transition. *Precambrian Res.* **124**, 69–85 (2003).
24. Cox, G. M. *et al.* Continental flood basalt weathering as a trigger for Neoproterozoic

Snowball Earth. *Earth Planet. Sci. Lett.* **446**, 89–99 (2016).

25. Bowyer, F. T. *et al.* Calibrating the temporal and spatial dynamics of the Ediacaran - Cambrian radiation of animals. *Earth-Science Rev.* **225**, 103913 (2022).
26. Dodd, M. S. *et al.* Development of carbonate-associated phosphate (CAP) as a proxy for reconstructing ancient ocean phosphate levels. *Geochim. Cosmochim. Acta* **301**, 48–69 (2021).
27. Rudnick, R. L. & Gao, S. Composition of the Continental Crust. in *Treatise on Geochemistry* vol. 4 1–51 (Elsevier, 2014).
28. Zhang, K., Tarbuck, G. & Shields, G. A. Refining the carbonate-associated iodine redox proxy with leaching experiments. *Chem. Geol.* **646**, 121896 (2024).
29. Brand, U. & Veizer, J. Chemical Diagenesis of a Multicomponent Carbonate System--1: Trace Elements. *J. Sediment. Res.* **50**, 987–998 (1980).
30. Banner, J. L. & Hanson, G. N. Calculation of simultaneous isotopic and trace element variations during water-rock interaction with applications to carbonate diagenesis. *Geochim. Cosmochim. Acta* **54**, 3123–3137 (1990).
31. Kaufman, A. J. & Knoll, A. H. Neoproterozoic variations in the C-isotopic composition of seawater: stratigraphic and biogeochemical implications. *Precambrian Res.* **73**, 27–49 (1995).
32. Romaniello, S. J., Herrmann, A. D. & Anbar, A. D. Uranium concentrations and 238U/235U isotope ratios in modern carbonates from the Bahamas: Assessing a novel paleoredox proxy. *Chem. Geol.* **362**, 305–316 (2013).
33. Hardisty, D. S. *et al.* Perspectives on Proterozoic surface ocean redox from iodine contents in ancient and recent carbonate. *Earth Planet. Sci. Lett.* **463**, 159–170 (2017).
34. Lau, K. V. & Hardisty, D. S. Modeling the impacts of diagenesis on carbonate paleoredox proxies. *Geochim. Cosmochim. Acta* **337**, 123–139 (2022).
35. Kamber, B. S., Webb, G. E. & Gallagher, M. The rare earth element signal in Archaean microbial carbonate: Information on ocean redox and biogenicity. *J. Geol. Soc. London.* **171**, 745–763 (2014).
36. Tostevin, R. Cerium Anomalies and Paleoredox. in *Elements in Geochemical Tracers in Earth System Science* (Cambridge University Press, 2021). doi:10.1017/9781108847223.

37. Zhang, K. & Shields, G. A. Early diagenetic mobilization of rare earth elements and implications for the Ce anomaly as a redox proxy. *Chem. Geol.* **635**, 121619 (2023).

38. Goto, K. T. *et al.* Uranium isotope systematics of ferromanganese crusts in the Pacific Ocean: Implications for the marine 238U/235U isotope system. *Geochim. Cosmochim. Acta* **146**, 43–58 (2014).

39. Abshire, M. L. *et al.* Uranium isotopes as a proxy for primary depositional redox conditions in organic-rich marine systems. *Earth Planet. Sci. Lett.* **529**, 115878 (2020).

40. Clarkson, M. O. *et al.* Upper limits on the extent of seafloor anoxia during the PETM from uranium isotopes. *Nat. Commun.* **12**, 1–9 (2021).

41. Chen, X., Robinson, S. A., Romaniello, S. J. & Anbar, A. D. 238U/235U in calcite is more susceptible to carbonate diagenesis. *Geochim. Cosmochim. Acta* **326**, 273–287 (2022).

42. Somlyay, A. *et al.* Uranium isotope evidence for extensive seafloor anoxia after the end-Triassic mass extinction. *Earth Planet. Sci. Lett.* **614**, 118190 (2023).

43. Zhang, Y. *et al.* The isotopic composition of sedimentary organic zinc and implications for the global Zn isotope mass balance. *Geochim. Cosmochim. Acta* **314**, 16–26 (2021).

44. He, Z., Archer, C., Yang, S. & Vance, D. Sedimentary cycling of zinc and nickel and their isotopes on an upwelling margin: Implications for oceanic budgets and paleoenvironment proxies. *Geochim. Cosmochim. Acta* **343**, 84–97 (2023).

45. Dodd, M. S. *et al.* Uncovering the Ediacaran phosphorus cycle. *Nature* **618**, 974–980 (2023).

46. Lyons, T. W., Walter, L. M., Gellatly, A. M., Martini, A. M. & Blake, R. E. Sites of anomalous organic remineralization in the carbonate sediments of South Florida, USA: The sulfur cycle and carbonate-associated sulfate. *Spec. Pap. Geol. Soc. Am.* **379**, 161–176 (2004).

47. Gill, B. C., Lyons, T. W. & Frank, T. D. Behavior of carbonate-associated sulfate during meteoric diagenesis and implications for the sulfur isotope paleoproxy. *Geochim. Cosmochim. Acta* **72**, 4699–4711 (2008).

48. Rennie, V. C. F. & Turchyn, A. V. The preservation of δSSO434 and δOSO418 in carbonate-associated sulfate during marine diagenesis: A 25 Myr test case using

marine sediments. *Earth Planet. Sci. Lett.* **395**, 13–23 (2014).

49. Present, T. M. *et al.* Diagenetic controls on the isotopic composition of carbonate-associated sulphate in the Permian Capitan Reef Complex, West Texas. *Sedimentology* **66**, 2605–2626 (2019).
50. Schurr, S. L., Strauss, H., Mueller, M. & Immenhauser, A. Assessing the robustness of carbonate-associated sulfate during hydrothermal dolomitization of the Latemar platform, Italy. *Terra Nov.* **33**, 621–629 (2021).
51. Present, T. M., Adkins, J. F. & Fischer, W. W. Variability in Sulfur Isotope Records of Phanerozoic Seawater Sulfate. *Geophys. Res. Lett.* **47**, 1–17 (2020).
52. Present, T. M., Paris, G., Burke, A., Fischer, W. W. & Adkins, J. F. Large Carbonate Associated Sulfate isotopic variability between brachiopods, micrite, and other sedimentary components in Late Ordovician strata. *Earth Planet. Sci. Lett.* **432**, 187–198 (2015).
53. Johnson, D. L., Present, T. M., Li, M., Shen, Y. & Adkins, J. F. Carbonate associated sulfate (CAS) δ34S heterogeneity across the End-Permian Mass Extinction in South China. *Earth Planet. Sci. Lett.* **574**, 117172 (2021).
54. Canfield, D. E. Biogeochemistry of Sulfur Isotopes. *Rev. Mineral. Geochemistry* **43**, 607–636 (2001).
55. Habicht, K. S., Gade, M., Thamdrup, B., Berg, P. & Canfield, D. E. Calibration of sulfate levels in the Archean ocean. *Science* **298**, 2372–2374 (2002).
56. Wang, P. *et al.* Large accumulations of ^{34}S-enriched pyrite in a low-sulfate marine basin: The Sturtian Nanhua Basin, South China. *Precambrian Res.* **335**, 105504 (2019).
57. Hurtgen, M. T., Arthur, M. A., Suits, N. S. & Kaufman, A. J. The sulfur isotopic composition of Neoproterozoic seawater sulfate: Implications for a snowball Earth? *Earth Planet. Sci. Lett.* **203**, 413–429 (2002).
58. Marenco, P. J., Corsetti, F. A., Hammond, D. E., Kaufman, A. J. & Bottjer, D. J. Oxidation of pyrite during extraction of carbonate associated sulfate. *Chem. Geol.* **247**, 124–132 (2008).
59. Edwards, C. T., Fike, D. A. & Saltzman, M. R. Testing carbonate-associated sulfate (CAS) extraction methods for sulfur isotope stratigraphy: A case study of a Lower–Middle Ordovician carbonate succession, Shingle Pass, Nevada, USA. *Chem. Geol.*

529, 119297 (2019).

60. Gorjan, P., Veevers, J. J. & Walter, M. R. Neoproterozoic sulfur-isotope variation in Australia and global implications. *Precambrian Res.* **100**, 151–179 (2000).

61. Herrmann, A. D., Gordon, G. W. & Anbar, A. D. Uranium isotope variations in a dolomitized Jurassic carbonate platform (Tithonian; Franconian Alb, Southern Germany). *Chem. Geol.* **497**, 41–53 (2018).

62. Vance, D. *et al.* The oceanic budgets of nickel and zinc isotopes: the importance of sulfidic environments as illustrated by the Black Sea. *Philos. Trans. R. Soc. A Math. Phys. Eng. Sci.* **374**, 20150294 (2016).

63. Parnell, J. & Boyce, A. J. Microbial sulphate reduction during Neoproterozoic glaciation, Port Askaig Formation, UK. *J. Geol. Soc. London.* **174**, 850–854 (2017).

64. Zhang, F. *et al.* Uranium isotope evidence for extensive shallow water anoxia in the early Tonian oceans. *Earth Planet. Sci. Lett.* **583**, 117437 (2022).

65. Lau, K. V., Lyons, T. W. & Maher, K. Uranium reduction and isotopic fractionation in reducing sediments: Insights from reactive transport modeling. *Geochim. Cosmochim. Acta* **287**, 65–92 (2020).

66. Chen, X. *et al.* Anoxic depositional overprinting of 238U/235U in calcite: When do carbonates tell black shale tales? *Geology* **49**, 1193–1197 (2021).

67. Anderson, R. F., Fleisher, M. Q. & LeHuray, A. P. Concentration, oxidation state, and particulate flux of uranium in the Black Sea. *Geochim. Cosmochim. Acta* **53**, 2215–2224 (1989).

68. Tissot, F. L. H. *et al.* Controls of eustasy and diagenesis on the 238U/235U of carbonates and evolution of the seawater (234U/238U) during the last 1.4 Myr. *Geochim. Cosmochim. Acta* **242**, 233–265 (2018).

69. Clarkson, M. O. *et al.* Environmental controls on very high $\delta^{238}U$ values in reducing sediments: Implications for Neoproterozoic seawater records. *Earth-Science Rev.* **237**, 104306 (2023).

70. Johnston, D. T., Macdonald, F. A., Gill, B. C., Hoffman, P. F. & Schrag, D. P. Uncovering the Neoproterozoic carbon cycle. *Nature* **483**, 320–323 (2012).

71. Cao, W., Lee, C. T. A. & Lackey, J. S. Episodic nature of continental arc activity since 750 Ma: A global compilation. *Earth Planet. Sci. Lett.* **461**, 85–95 (2017).

72. Dutkiewicz, A. *et al.* Duration of Sturtian “Snowball Earth” glaciation linked to

exceptionally low mid-ocean ridge outgassing. *Geology* **52**, 292–296 (2024).

73. Müller, R. D. *et al.* Evolution of Earth's tectonic carbon conveyor belt. *Nature* **605**, 629–639 (2022).
