# Supplementary material for: Ocean deoxygenation after the Sturtian Snowball
Source: Nat Commun. 2025 Jul 1;16:5618. doi: 10.1038/s41467-025-60700-w (PMC12219592; doi:10.1038/s41467-025-60700-w)
Supplement: Supplementary file 2 — Description of Additional Supplementary Files [file 41467_2025_60700_MOESM2_ESM.docx]

**Description of Additional Supplementary Files:**

**Supplementary Data 1.** Geochemical data for the studied samples.
